# Canine Mammary Tumors as a Potential Model for Human Breast Cancer in Comparative Oncology

**DOI:** 10.1155/2024/9319651

**Published:** 2024-05-10

**Authors:** Amirhossein Razavirad, Sanaz Rismanchi, Pejman Mortazavi, Ahad Muhammadnejad

**Affiliations:** ^1^Cancer Biology Research Center, Cancer Institute of Iran, Tehran University of Medical Sciences, Tehran, Iran; ^2^Department of Pathobiology, Science and Research Branch, Islamic Azad University, Tehran, Iran

## Abstract

Clinical and molecular similarities between canine mammary tumors (CMTs) and human breast cancer (HBC) propel scientists to further study their application in comparative oncology as a model for human breast cancer. In total, 64 canine mammary tumors were selected to study the most common markers, which are applicable for human breast cancer treatment, including estrogen and progesterone receptors (ER and PR), human epidermal growth factor (HER2/neu), Ki67, and cyclooxygenase 2 (Cox2). Immunohistochemistry (IHC) was used to assess the protein expression. The Veterinary Nottingham Prognostic Index (Vet-NPI) was also computed. Moreover, univariate and multivariable Cox proportional hazard analyses were applied to estimate hazard ratios (HRs). The results demonstrated that Ki67 was strongly expressed in the triple-negative tumors, and Ki67 protein expression continuously increased over the increase of Cox2 protein expression (*p* < 0.001). Further analysis revealed a significant difference among canine mammary subtypes and Vet-NPI, in which triple-negative tumors displayed the highest mean score compared to other subtypes (*p* < 0.001). In addition, the multivariable analysis revealed that the regional mastectomy procedure (adjusted HR = 2.78 (1.14–6.8)), the triple-negative tumors (adjusted HR = 48.08 (7.74–298.8)), strong Ki67 protein expression group (adjusted HR = 7.88 (2.02–30.68)), and strong Cox2 protein expression group (adjusted HR = 29.35 (5.18–166.4)) demonstrated significantly lower disease-free survival rates compared to other corresponding groups. Overall, canine mammary tumors showed strong similarities to human breast cancer in terms of clinical and molecular aspects; therefore, they could be suggested as a model for human breast cancer in comparative oncology.

## 1. Background

Despite the progress in human breast cancer (HBC) research and treatment, their incidence is increasing by 0.5% annually; therefore, discovering new treatment strategies seems crucial [1]. Although mouse laboratory models have significantly contributed to HBC research, traditional laboratory mouse models do not spontaneously develop tumors. Thus, genetically engineered or patient-derived xenograft (PDX) models are often required for more complex HBC studies [2]. Given that canine mammary tumors (CMTs) arise spontaneously and share other characteristics with HBC, such as incidence rate, age at onset, hormonal influence, subtype classification, and disease course [2, 3], these models can therefore become an excellent alternative to human clinical studies [4].

In mammals, ovarian steroids, including estrogen and progesterone, affect the mammary epithelium, which can lead to malignant proliferation [5]. The steroid hormones exert their biological effects via specific receptors, including estrogen and progesterone receptors (ER and PR) [6]. There are two known isoforms of the ERs, ER*α* and ERß [7]. The role of ER*α* in human breast cancer and canine mammary tumors is well known; however, further research on the role of ERß is still required [8]. Like HBC, the presence of ER*α* in CMT has been associated with pathological disease features and tumoral differentiation [9]. In addition, the progesterone receptor (PR) plays a vital role in the growth of mammary glands. It causes the expansion of the lobular units of the terminal ductal during puberty and pregnancy [10].

Over the past few decades, some molecular markers associated with tumor growth and development have been recognized in humans and canines. These include human epidermal growth factor receptor 2 (HER2/neu), Ki67, and cyclooxygenase 2 (Cox2) [11–13]. HER2/neu is a proto-oncogene widely studied in human medicine and has shown extraordinary therapeutic results in response to anti-HER2 drugs, such as trastuzumab [14, 15]. Ki67 is a nuclear protein more expressed in the cell cycle and is reported as a marker for proliferation [16]. There is credible evidence suggesting the role of Cox2 in tumor growth and development in both species [13, 17]. Researchers believe that the upregulation of Cox2 in human breast cancer is involved in prostaglandin production during tumorigenesis. Therefore, it could be a significant target for nonsteroid anti-inflammatory medicines, such as celecoxib [18].

It is worth mentioning that there are still gaps in the research, including the need to determine the impact of surgical procedures on treatment outcomes and the ability to classify mammary tumors into distinct subtypes similar to HBC [2, 19–21]. The present study attempted to determine the molecular subtypes of canine mammary tumors according to ER*α*, PR, and HER2 statuses and measure the protein expression of Ki67 and Cox2 to compare the similarities and differences between the two species. In addition, the disease-free survival (DFS, the time from surgical resection to tumor recurrence or metastasis) was computed by accurately recording clinical, surgical, and histopathological data.

## 2. Materials and Methods

### 2.1. Animals and Tumors

Sixty-four spontaneous CMTs from veterinary clinics and hospitals in Tehran, Iran, were prospectively studied. The histological classification of CMTs includes malignant epithelial neoplasms, malignant epithelial neoplasms of special types, malignant mesenchymal neoplasms-sarcoma, carcinosarcoma-malignant mixed mammary tumors, benign neoplasms, hyperplasia/dysplasia, neoplasms of the nipple, and hyperplasia/dysplasia of the nipple [22]. All the specimens used in our study were diagnosed as (1) malignant epithelial neoplasms, such as (a) simple carcinoma, (b) micropapillary carcinoma, (c) complex carcinoma, (d) complex-mix carcinoma, (e) solid carcinoma, (f) comedo carcinoma, (g) anaplastic carcinoma, (h) malignant myoepithelioma, and (i) intraductal papillary carcinoma, or (2) malignant epithelial neoplasms of special type, such as (a) squamous cell carcinoma, (b) adenosquamous carcinoma, and (c) spindle carcinoma. Informed consent was received from animal owners, and the study was approved by the Ethics Committee of Islamic Azad University of Tehran, Iran–Science & Research Branch (No. IR.IAU.SRB.REC.1398.180). The median age of the animals was 10 years, ranging from 5 to 15 years. As presented in Table 1, clinicopathological features, such as age, tumor laterality, tumor size, type of surgery, lymph node status, metastasis, histological grade, and TNM staging, were available for the study. Tumor size was classified according to the WHO criteria (≤3 cm, 3–5 cm, and ≥5 cm). The surgical procedures were categorized into three types [19]. The first type was simple mastectomy (36 cases), which included removing the affected gland only. The second type was regional mastectomy (20 cases), which included removing the affected gland and glands that share lymphatic drainage along with associated lymph nodes. The third type was radical mastectomy (8 cases), which included removing the entire mammary chain and associated lymph nodes either unilaterally or bilaterally. According to the TNM staging classification, 31 animals were in stage I, 20 were in stage II, and 13 were in stage III. In addition, 27 tumors were classified as grade 1, 25 as grade 2, and 12 as grade 3. Veterinary Nottingham Prognostic Index (Vet-NPI) was taken from the study of Haybittle et al. [23] and computed as follows: Vet-NPI = [tumor size (cm) × 0.2] + histological grade (1, 2, or 3) + evidence of vascular invasion/regional lymph node metastases (1 or 2 if absent or present, respectively). As previously described, Vet-NPI was classified into good, moderate, and poor [24].

### 2.2. Follow-Up Study

Animals were followed up for 24 months after surgery, and their disease-free survival (DFS) was computed.

### 2.3. Sample Preparation and Immunohistochemistry (IHC)

All samples were fixed in formalin embedded in paraffin and kept at the Cancer Biology Research Center of the Cancer Institute of Iran, Imam Khomeini Hospital Complex, Tehran University of Medical Sciences, the I.R. of Iran, from April 2010 to February 2020. Briefly, the samples were subsequently sliced and processed to the thickness of 3 *µ*m for IHC. The tissue sections were placed in the oven for 40 minutes, immediately immersed in xylene to remove residual paraffin, and then hydrated by gradient alcohol. The slides were boiled in citrate buffer (pH = 6.0) with 10% to 20% reduced power to retrieve the antigens for 20 minutes and incubated at room temperature for 30 minutes. After exhausting endogenous peroxidase using H_2_O_2_ in methanol for 15 minutes, the sections were rinsed three times with phosphate-buffered saline (PBS) and then blocked with 5% bovine serum albumin (BSA) at room temperature for one hour. The sections were rinsed three times in PBS, incubated with specific antibodies at room temperature for one hour, and rinsed with PBS thrice. The sections were incubated in horseradish peroxidase (HRP), rinsed thrice with PBS, and counterstained by the Harris hematoxylin method. Primary antibodies against ER*α* (mouse monoclonal, orb389114, dilution 1 : 100), PR (rabbit polyclonal, orb106338, dilution 1 : 100), and HER2 (rabbit polyclonal, orb315778, dilution 1 : 100) were purchased from Biorbyt (Cambridge, UK). In addition, Ki67 (rabbit monoclonal, SKU: 325, ready-to-use) and Cox2 (rabbit monoclonal, SKU: 306, dilution 1 : 50) were purchased from Biocare Medical (CA, USA).

### 2.4. Molecular Subtypes of Canine Mammary Tumors

The expression of ER*α* and PR protein was quantified based on the percentage of cells with nuclear positivity. HER2 protein expression was measured according to the percentage of cells with uniform intense complete membrane staining. We classified canine mammary tumors into different subtypes as previously described [25]. ER-positive, PR-positive, and HER2-negative were defined as luminal A tumors. ER-positive, PR-positive, and HER2-positive were defined as luminal B tumors. ER-negative, PR-negative, and HER2-negative were defined as triple-negative tumors. ER-negative, PR-negative, and HER2-positive were defined as HER2-enriched tumors.

### 2.5. Immunoreactivity Scoring Method

**Figure 1:**
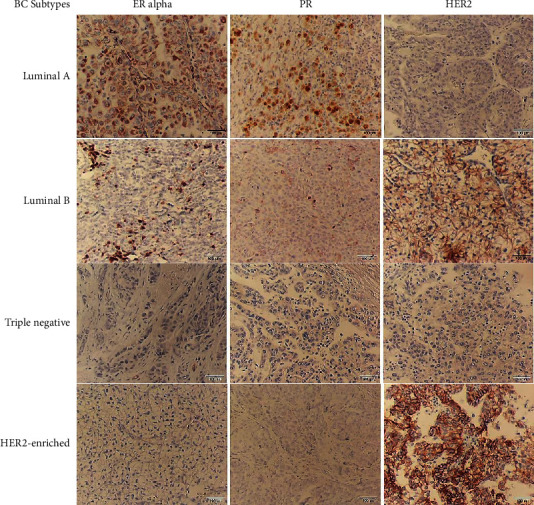
Representative images of CMT subtypes by IHC. ER*α* and PR protein expression was measured according to the percentage of cells with nuclear positivity. HER2 protein expression was quantified based on the percentage of cells with uniform intense complete membrane staining. The labeled images indicate subtypes of canine mammary tumors, including luminal A, luminal B triple-negative, and HER2-enriched. Scale bars are equal to 100 *µ*m.

**Figure 2:**
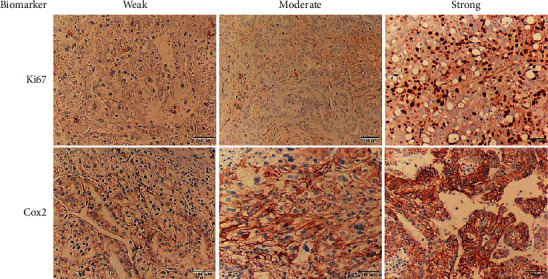
Representative images of Ki67 and Cox2 staining in canine mammary tumors by IHC. Ki67 protein expression was quantified based on the percentage of cells with positive nuclei. Cox2 protein expression was measured according to the proportion and intensity of cytoplasmic staining. Canine mammary adenocarcinomas with weak, moderate, and strong Ki67 staining as well as weak, moderate, and strong Cox2 staining. Scale bars are equal to 100 *µ*m.

As shown in Figures 1 and 2, all formalin-fixed paraffin-embedded (FFPE) tissue sections were reviewed by an expert pathologist. The expression of ER and PR was measured based on the percentage of positive nuclear cells, where a score <1 was considered negative, from 1 to 10 as weak, and >10 as positive protein expression [26]. In addition, HER2 equal to 0 or +1 was considered negative, +2 as equivocal, and +3 as a positive expression [26]. Ki67 ranged from 1 to 43 (in percentage) and was quantified by scoring the nuclear staining intensity, where a score of <5 was considered weak, from 5 to 14 as moderate, and ≥15 as strong expression [25]. Additionally, Cox2 status was determined according to the modified Allred scoring system, which is a combination of two parameters, including the proportion and intensity scores, where scores 0 and 1 were considered as unexpressed, 2 and 3 as weak, from 4 to 5 as moderate, and from 6 to 8 as strong expression [27].

### 2.6. Statistical Analysis

One-way ANOVA was computed to compare a continuous variable with categorical explanatory variables. All values were expressed as mean and standard deviation (±SD). Tukey's post hoc test with a 95% confidence interval (CI) was performed to evaluate the association between different variables in pairs. The Kaplan–Meier method was applied for survival analysis, and the log-rank test calculated the differences. The Cox proportional hazard model was employed for the univariate and multivariate survival analysis. In all statistical analyses, *p* < 0.05 was considered significant. The RStudio statistical software version 1.2.5033 was used for statistical analysis.

## 3. Results

### 3.1. Canine Mammary Tumors Exhibited Various Clinical Features and Protein Expression Patterns

**Figure 3:**
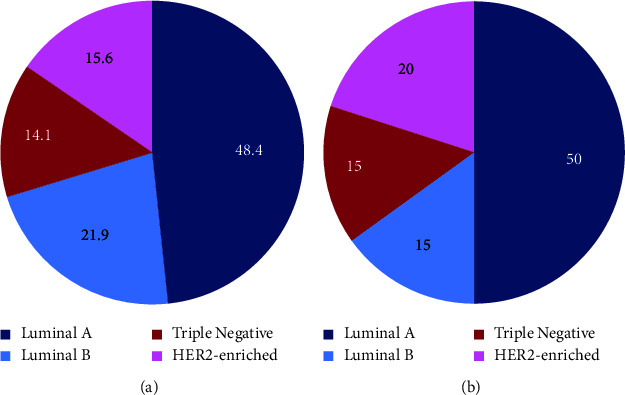
Pie charts of subtype frequencies comparing canine mammary tumors and human breast cancer. Pie charts show the frequency of subtypes in canine mammary tumors and human breast cancer that are nearly equal. (a) Frequency (%) of CMT subtypes in our study. (b) Frequency (%) of human breast cancer subtypes.

Animals ranged in age from 5 to 15 years with a mean and standard deviation (±SD) of 9.78 ± 2.7 years. Tumor size varied from 0.7 to 7.3 cm with a mean (±SD) of 3.01 ± 1.56 cm. Vet-NPI ranged from 1.14 to 6.46 with a mean (±SD) of 3.13 (±1.57). All 64 canine mammary tumors were classified into subtypes based on ER*α*, PR, and HER2 protein expression. The expression of ER*α* and PR protein was quantified based on the percentage of cells with nuclear positivity. For ER*α*, 19/64^*∗*^100 = 29.69% tumors were negative, 8 (12.5%) were weak, and 37 (57.81%) were positive. In addition, for PR, 30 (46.87%) tumors were negative, 5 (7.81%) were weak, and 29 (45.32%) were positive. Additionally, HER2 protein expression was quantified according to the percentage of cells with uniform intense complete membrane staining, where 45 (70.31%) tumors were considered negative, and 19 (29.69%) were considered positive. After subtyping mammary tumors, 31 (48.4%) tumors were classified as luminal A, 14 (21.9%) as luminal B, 9 (14.1%) as triple-negative, and 10 (15.6%) as HER2-enriched (Table 2 and Figure 3(a)). Based on Ki67 protein expression evaluation, 14 tumors (21.9%) were classified as weak, 21 tumors (32.8%) as moderate, and 29 tumors (45.3%) as strong expression (Table 2). In addition, evaluation of Cox2 protein expression indicated that 26 tumors (40.6%) were negative, 15 tumors (23.4%) were weak, 14 tumors (21.9%) were moderate, and nine tumors (14.1%) were strong (Table 2).

### 3.2. Triple-Negative Tumors Displayed Distinct Characteristics Compared with Other Tumor Subtypes

**Figure 4:**
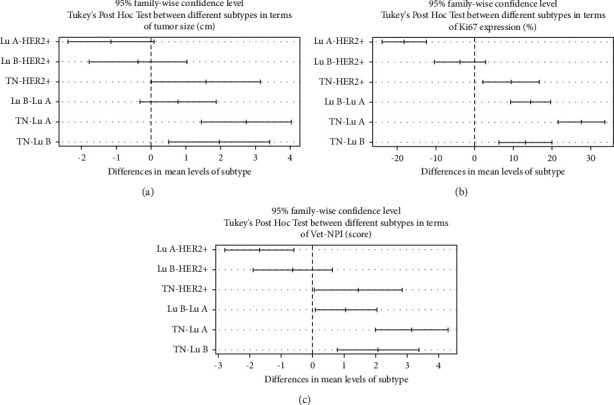
Tukey's post hoc test with 95% confidence interval. Tukey's post hoc test with a 95% confidence interval is conducted to evaluate the correlation between different subtypes with tumor size in centimeter (a), Ki67 in percentage (b), and Vet-NPI (c).

One-way ANOVA revealed a significant difference between canine mammary subtypes and tumor size, in which triple-negative tumors possessed the highest mean tumor size compared to other subtypes (*p* < 0.001, Table 3). The same analysis was performed between canine mammary subtypes and Ki67 protein expression so that triple-negative tumors exhibited the highest expression among different subtypes (*p* < 0.001, Table 3). Additionally, further analysis revealed a significant difference between canine mammary subtypes and Vet-NPI, in which triple-negative tumors displayed the highest mean score compared to other subtypes (*p* < 0.001, Table 3). To provide a clearer picture of the differences between different subtypes in relation to tumor size, Cox2 status, and Vet-NPI, a Tukey's post hoc test was performed (Figures 4(a)–4(c)). This analysis revealed that the differences in tumor size were significant only between triple-negative tumors with luminal A, luminal B, and HER2-enriched subtypes (Figure 4(a)). However, for Cox2 and Vet-NPI statuses, the differences were significant for all subtypes, except luminal B and HER2-enriched pairwise subtypes (Figures 4(b) and 4(c)). Likewise, one-way ANOVA was performed on tumor size, Ki67 protein expression, and Vet-NPI status, comparing them with tumors expressing Cox2 differentially. The results suggested a significant trend between these variables and Cox2 protein expression from negative to strong expression statuses (*p* < 0.001, Table 4).

### 3.3. Multivariable Analysis Indicated the Worst DFS for Triplenegative, Ki67-strong, and Cox2-Strong Tumors Compared to Other Relevant Subgroups

**Figure 5:**
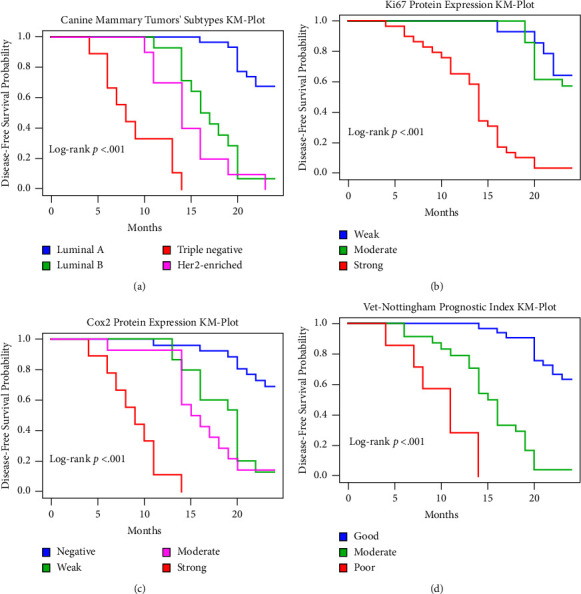
Kaplan–Meier plots indicating disease-free survival probabilities among 64 available canine mammary tumors. (a) Dogs having triple-negative (TN) tumors demonstrate significantly poor disease-free survival compared with other subtypes of canine mammary tumors (log-rank *p* < 0.001). (b, c) Tumors expressing high Ki67 and Cox2 protein displayed significantly poor disease-free survival compared with other tumor subgroups (log-rank *p* < 0.001). (d) Dogs presenting poor Vet-NPI indicated significantly poor disease-free survival compared with other groups (log-rank *p* < 0.001). For each Kaplan–Meier plot, a corresponding log-rank *p* value was presented.

Our univariate analysis showed that tumors greater than 5 cm, vascular invasion involvement, perineural invasion involvement, necrosis presence, histological grade 3, TNM stage III, radical mastectomy procedure, triple-negative subtype, strong Ki67, and strong Cox2 exhibited significantly the worst DFS compared to their corresponding subgroups (Table 2). Multivariable analysis adjusted with age, vascular invasion, perineural invasion, histological grade, and TNM stage for each marker individually revealed that the regional mastectomy procedure, the triple-negative tumors, strong Ki67 protein expression group, and strong Cox2 protein expression group demonstrated significantly lower disease-free survival rates compared with other corresponding subgroups (Table 2). Survival curves depicted by Kaplan–Meier plots and relevant log-rank *p* values supported the multivariable analysis, where the triple-negative tumors, strong Ki67 protein expression, and strong Cox2 protein expression exhibited the worst disease-free survival rates compared to the relevant groups (log-rank *p* value <0.001, Figures 5(a)–5(c)). Tumors with the highest Vet-NPI score, classified as a poor group, exhibited the lowest disease-free survival rate compared to others (Figure 5(d)).

## 4. Discussion

Mammary tumors are the most common type of tumor in intact female dogs, accounting for around 42% of all tumors [28, 29]. Recently, it has become increasingly clear that canine mammary tumors are clinically and molecularly similar to human breast cancer [30–32]. Here, we discuss some clinical, pathological, and molecular similarities in both species, suggesting that CMT can be used as a reliable model for human breast cancer in comparative oncology.

Research indicates that despite having different lifespans, the average age of onset at which dogs are diagnosed with mammary tumors (after six years) is very similar to that of women (after 40 years) [33]. In both species, tumor size is directly related to survival and has an independent prognostic value [31, 34, 35]. Patients with non-necrotic tumors, without vascular and perineural invasions, with well-differentiated tumor cells, and in the early stages experience significantly longer survival after primary mammary tumor surgery [36]. Consistent with these findings, our cohort indicated that the average age of onset is after six years, and tumors larger than 3 cm have the significantly lowest DFS compared to other relevant subgroups. We also found that the presence of vascular and perineural invasion, poorly differentiated tumors, and advanced stages are of prognostic value for DFS. These variables are significantly associated with poor prognosis and low DFS. Hörnfeldt and Mortensen reviewed twelve studies on surgical dose and clinical outcome in the treatment of mammary gland tumors in female dogs. According to their analysis, no study shows a clear advantage in choosing one surgical method over the other [19]. However, our study, with its potential to significantly influence future research in this area, has uncovered significant findings. We found that the dogs that underwent radical mastectomy had a significantly lower DFS rate than dogs that received other relevant surgical procedures. Moreover, our multivariable analysis indicated that the regional mastectomy method could be an independent prognostic factor. These findings suggest that surgical procedures can be proposed as prognostic factors and help fill the gaps in this field. The choice of surgical procedure depends on the tumor size (pT), which can predict lymph node involvement. Therefore, a large sample size with various surgical procedure designs is necessary to compare the efficacy of treatment outcomes.

In recent years, extensive veterinary research has been conducted on tumor markers, such as ER*α*, PR, HER2, Ki67, and Cox [37–41]. Reports confirm the unique similarities between human breast cancer and canine mammary tumors regarding these markers' protein expression and prognostic values [42]. ER*α* is upregulated in two-thirds of breast tumors and can influence endocrine therapy in terms of treatment selection or response to treatment [43]. Therefore, ER*α* status can be considered a valuable prognostic indicator in CMT. We reported that the reduced expression of ER*α* is associated with the aggressive phenotype of the tumor and poor prognosis [42]. In addition, decreased PR expression is associated with a poor prognosis and can be a reliable indicator of recurrence [44]. The combined status of ER*α*, PR, and HER2/neu indicated differences in prognosis, whereas ER*α*-negative, PR-negative, and HER2/neu-negative tumors showed the worst prognosis [38, 45]. In line with these findings, we reported that triple-negative tumors (ER*α*-, PR-, and HER2-) exhibited less disease-free survival and more recurrence compared with other relevant subtypes. Additionally, given that some studies classify canine mammary tumors into four subtypes, including luminal A, luminal B, triple negative, and HER2-enriched [2, 20], others cannot classify HER2-enriched as an independent subtype [21]. Therefore, more studies can be helpful to accurate classification of mammary tumor subtypes. Our classification in this study was consistent with previous studies that classified canine mammary tumors into four subtypes. Furthermore, as shown in Figure 3, the frequency of subtypes in our study was almost similar to that in human breast cancer [46]. These results could further prove that CMT could be a potential model for human breast cancer in comparative oncology.

The Ki67 is a nuclear protein highly expressed in proliferating cells [47]. Although studies on Ki67 protein expression have used different cutoff points, most have shown a significant association between higher Ki67 protein expression and a worse prognosis [40, 48]. Similarly, we found comparable results indicating a correlation between higher Ki67 expression and a poor prognosis. In addition, human breast studies have revealed that Cox2 overexpression plays a crucial role in prostaglandin production over tumorigenesis and is involved in the early steps of mammary carcinogenesis [49, 50]. Studies on CMTs show a significant relationship between higher levels of Cox2 expression and decreased DFS [13, 41]. Interestingly, we observed similar findings in our dataset, where higher Cox2 protein expression was significantly associated with higher Ki67 protein expression and reduced DFS. Most recently, our group in a case series investigated the expression of Cox2 in canine inflammatory mammary carcinoma and concluded that this model could be suitable for comparative oncology [51].

This study has potential limitations, such as limited sample size and lack of information on the breed, spaying status, diet, environmental factors, and comorbidities. These confounding factors may affect prognosis and survival. In addition, due to the limited sample size in each subgroup of histological classification, we could not correlate them with protein expression and clinicopathological parameters. Hence, a larger sample size with functional or genetic analysis of the tumor, including gene expression, mutation, or copy number variation profiles, can provide more insights into the molecular mechanisms and pathways involved in the disease. Additionally, the study does not evaluate the response or resistance of the canine mammary tumors to any specific treatments, such as chemotherapy, hormonal therapy, or targeted therapy, which could determine the suitability and translatability of the canine model for human breast cancer therapy.

Overall, the cumulative similarities presented here strengthen the hypothesis of considering CMTs as a source for increasing the understanding of HBC molecular pathogenesis. Despite the many similarities identified between CMT and HBC that propose it as a reliable model in comparative oncology, limitations include high cost, being time-consuming, and reliance on dog owners in postoperative care. Nevertheless, this model can be used in human breast clinical trials to develop novel therapeutic strategies in comparative oncology.

## Figures and Tables

**Table 1:** Clinical and para-clinical data of 64 canine mammary tumors hospitalized in veterinary clinics and hospitals in Tehran during 2010–2020.

Variable	No.	Frequency (%)
*Age (years)*		
≤8	23	35.94
9–11	21	32.81
≥12	20	31.25

*Tumor size (cm)*		
<3	31	48.44
3.1–4.9	26	40.62
≥5	7	10.94

*Laterality*		
Right	28	43.75
Left	36	56.25

*Lymph node status*		
N0	50	78.12
N1	14	21.88

*Vascular invasion*		
Absent	29	45.31
Present	35	54.69

*Perineural invasion*		
Absent	51	79.69
Present	13	20.31

*Necrosis*		
Absent	39	60.94
Present	25	39.06

*Type of surgery*		
Simple mastectomy	36	56.25
Regional mastectomy	20	31.25
Radical mastectomy	8	12.5

*Histological grade*		
Grade 1	27	42.19
Grade 2	25	39.06
Grade 3	12	18.75

*TNM stage*		
Stage I	31	48.44
Stage II	20	31.25
Stage III	13	20.31

**Table 2:** Univariate and multivariable analyses of prognostic factors for 64 available canine mammary tumors.

Variable	Number (%)		Univariate	Multivariable
	Cases (*n* = 64)	DFS (*n* = 22)	HR (95% CI)	Adjusted HR (95% CI)
*Age (years)*				
≤8	23 (35.9)	5 (21.7)	1	—
9–11	21 (32.8)	8 (38.1)	0.69 (0.34–1.42)	
≥12	20 (31.3)	9 (45.0)	0.59 (0.28–1.25)	

*Tumor size*				
<3 cm	31 (48.4)	18 (58.1)	1	—
3–5 cm	26 (40.6)	3 (11.5)	4.11 (2.05–8.27)	
>5 cm	7 (11.0)	1 (14.3)	7.8 (2.92–20.81)	

*Vascular invasion*				
Absent	29 (45.3)	17 (58.6)	1	—
Present	35 (54.7)	5 (14.3)	4.36 (2.2–8.63)	

*Perineural invasion*				
Absent	51 (79.7)	22 (43.1)	1	—
Present	13 (20.3)	0 (0.0)	11.72 (5.14–26.7)	

*Necrosis*				
Absent	39 (60.9)	17 (43.59)	1	—
Present	25 (39.1)	5 (20.0)	2.17 (1.18–3.99)	

*Histological grade*				
Grade 1	27 (42.2)	18 (66.7)	1	—
Grade 2	25 (39.1)	4 (16.0)	5.55 (2.48–12.4)	
Grade 3	12 (18.7)	0 (0.0)	61.45 (18.71–201.8)	

*TNM stage*				
Stage I	31 (48.4)	18 (58.1)	1	—
Stage II	20 (31.3)	3 (15.0)	3.45 (1.65–7.2)	
Stage III	13 (20.3)	1 (7.7)	9.31 (4.1–21.15)	

*Type of surgery*				
Simple mastectomy	36 (56.25)	19 (52.78)	1	1
Regional mastectomy	20 (31.25)	2 (10)	3.59 (1.82–7.11)	2.78 (1.14–6.8)
Radical mastectomy	8 (12.5)	1 (12.5)	7.19 (2.93–17.68)	0.37 (0.07–1.88)

*Tumor subtypes*				
Luminal A	31 (48.4)	21 (67.7)	1	1
Luminal B	14 (21.9)	1 (7.1)	7.84 (3.31–18.57)	8.03 (2.94–21.94)
Triple negative	9 (14.1)	0 (0.0)	103.06 (30.83–344.52)	48.08 (7.74–298.8)
Her2-enriched	10 (15.6)	0 (0.0)	12.84 (5.17–31.93)	13.76 (4.31–43.94)

*Ki67 status*				
Weak	14 (21.9)	9 (64.3)	1	1
Moderate	21 (32.8)	12 (57.1)	1.31 (0.44–3.91)	0.83 (0.24–2.89)
Strong	29 (45.3)	1 (3.4)	13.02 (4.77–35.54)	7.88 (2.02–30.68)

*Cox2 status*				
Negative	26 (40.6)	18 (69.2)	1	1
Weak	15 (23.4)	2 (14.3)	5.04 (2.05–12.35)	3.74 (1.36–10.3)
Moderate	14 (21.9)	2 (13.3)	7.27 (2.9–18.24)	5.43 (1.86–15.87)
Strong	9 (14.1)	0 (0.0)	92.32 (26.56–320.9)	29.35 (5.18–166.4)

DFS, disease-free survival; HR, hazard ratio; CI, confidence interval. Adjusted HR: Cox proportional hazards ratio model fitted in each biomarker individually with age, vascular invasion, perineural invasion, histological grade, and TNM stage.

**Table 3:** Analyses of predictor variables in relation to subtypes of canine mammary tumors.

Variable	Tumor subtypes (mean ± SD)				*p* value (among groups)
	Luminal A	Luminal B	Triple negative	HER2-enriched	
Tumor size (cm)	2.28 ± 1.33	3.06 ± 1.24	5.01 ± 1.43	3.43 ± 1.05	<0.001
Ki67 expression	6.55 ± 3.94	21 ± 7.68	34.11 ± 5.75	24.7 ± 8.67	<0.001
Vet-NPI	2.2 ± 1.1	3.25 ± 1.39	5.34 ± 0.92	3.89 ± 1.2	<0.001

One-way ANOVA was applied to compute the *p* values among different subtypes of 64 canine mammary tumors. All variables displayed significant differences (*p* < 0.001), so that triple-negative tumors showed the highest mean compared with other subtypes. Veterinary Nottingham Prognostic Index (Vet-NPI) was computed as: Vet-NPI = [tumor size (cm) × 0.2] + histological grade (1, 2, or 3) + evidence of vascular invasion and/or regional lymph node metastases (1 or 2 if absent or present, respectively). SD, standard deviation.

**Table 4:** Analyses of predictor variables in relation to canine mammary tumors expressing differentially Cox2 protein.

Variable	Cox2 protein expression (mean ± SD)				*p* value (among groups)
	Negative	Weak	Moderate	Strong	
Tumor size (cm)	2.28 ± 1.31	2.9 ± 1.38	3.35 ± 1.44	4.8 ± 1.25	<0.001
Ki67 expression	8.11 ± 6.17	14.8 ± 11.98	23.86 ± 9.02	31.55 ± 6.17	<0.001
Vet-NPI	2.26 ± 1.19	3.11 ± 1.48	3.38 ± 1.29	5.29 ± 0.82	<0.001

One-way ANOVA was applied to compute the *p* values among 64 canine mammary tumors that differentially express Cox2 protein. All variables displayed significant differences (*p* < 0.001), so that triple-negative tumors showed the highest mean compared with other subgroups. Veterinary Nottingham Prognostic Index (Vet-NPI) was computed as: Vet-NPI = [tumor size (cm) × 0.2] + histological grade (1, 2 or 3) + evidence of vascular invasion and/or regional lymph node metastases (1 or 2 if absent or present, respectively). SD, standard deviation.

## Data Availability

The data that support the findings of this study are available from the corresponding author upon reasonable request.

## Abbreviations

CMT: Canine mammary tumor
HBC: Human breast cancer
ER: Estrogen receptor
PR: Progesterone receptor
HER2: Human epidermal growth factor receptor 2
Cox2: Cyclooxygenase 2
IHC: Immunohistochemistry
Vet-NPI: Veterinary Nottingham Prognostic Index
HR: Hazard ratio
PDX: Patient-derived xenograft
DFS: Disease-free survival
TNM: Tumor node metastasis
PBS: Phosphate-buffered saline
BSA: Bovine serum albumin
HRP: Horseradish peroxidase
FFPE: Formalin-fixed paraffin embedded
SD: Standard deviation
CI: Confidence interval
KM Plot: Kaplan–Meier Plot.
